# Fine-Mapping and Analysis of *Cgl1*, a Gene Conferring Glossy Trait in Cabbage (*Brassica oleracea* L. var. *capitata*)

**DOI:** 10.3389/fpls.2017.00239

**Published:** 2017-02-20

**Authors:** Zezhou Liu, Zhiyuan Fang, Mu Zhuang, Yangyong Zhang, Honghao Lv, Yumei Liu, Zhansheng Li, Peitian Sun, Jun Tang, Dongming Liu, Zhenxian Zhang, Limei Yang

**Affiliations:** ^1^Key Laboratory of Biology and Genetic Improvement of Horticultural Crops, Ministry of Agriculture, Institute of Vegetables and Flowers – Chinese Academy of Agricultural SciencesBeijing, China; ^2^College of Horticulture, China Agricultural UniversityBeijing, China

**Keywords:** cabbage, glossy mutant, fine-map, *Cgl1*, breeding

## Abstract

Cuticular waxes covering the outer plant surface impart a whitish appearance. Wax-less cabbage mutant shows glossy in leaf surface and plays important roles in riching cabbage germplasm resources and breeding brilliant green cabbage. This is the first report describing the characterization and fine-mapping of a wax biosynthesis gene using a novel glossy *Brassica oleracea* mutant. In the present paper, we identified a glossy cabbage mutant (line10Q-961) with a brilliant green phenotype. Genetic analyses indicated that the glossy trait was controlled by a single recessive gene. Preliminary mapping results using an F_2_ population containing 189 recessive individuals revealed that the *Cgl1* gene was located at the end of chromosome C08. Several new markers closely linked to the target gene were designed according to the cabbage reference genome sequence. Another population of 1,172 recessive F_2_ individuals was used to fine-map the *Cgl1* gene to a 188.7-kb interval between the C08SSR61 simple sequence repeat marker and the end of chromosome C08. There were 33 genes located in this region. According to gene annotation and homology analyses, the *Bol018504* gene, which is a homolog of *CER1* in *Arabidopsis thaliana*, was the most likely candidate for the *Cgl1* gene. Its coding and promoter regions were sequenced, which indicated that the RNA splice site was altered because of a 2,722-bp insertion in the first intron of *Bol018504* in the glossy mutant. Based on the FGENESH 2.6 prediction and sequence alignments, the PLN02869 domain, which controls fatty aldehyde decarbonylase activity, was absent from the *Bol018504* gene of the 10Q-961 glossy mutant. We inferred that the inserted sequence in *Bol018504* may result in the glossy cabbage mutant. This study represents the first step toward the characterization of cuticular wax biosynthesis in *B. oleracea*, and may contribute to the breeding of new cabbage varieties exhibiting a brilliant green phenotype.

## Introduction

Cuticular waxes, which are composed of a range of lipid compounds, act as a hydrophobic layer and cover the outer surface of aerial plant tissues (Millar et al., 1999). To adapt to environmental changes, plants secrete waxes onto the surface or into the interior of cuticles to form the first barrier against ultraviolet radiation, plant pathogens, and insects (Mariani and Wolters-Arts, 2000; Kunst and Samuels, 2003). Cuticular waxes can reduce non-stomatal water evapotranspiration and increase water retention, as well as prevent pollen, dust, and air pollutants from falling onto plant surfaces (Kerstiens, 1996; Barthlott et al., 1998). Moreover, cuticular waxes affect various physiological functions, such as preventing fruit cracking and influencing morphological development and pigmentation of leaves and fruits. Additionally, cuticular waxes regulate plant fertility by affecting pollen development (Koch and Ensikat, 2008; Koch et al., 2009).

Since Dellaert (Dellaert et al., 1979) discovered the first waxy cuticle eceriferum mutant (*cer*) in *Arabidopsis thaliana*, numerous genes involving in wax biosynthesis, export and regulation have hitherto been identified. In *A. thaliana, ABCG11, CER1, CER3, CER4*, CER5, *CER6*, CER7, *CER10, CFL1, FATB, HDG1, KCR1, LACS1, LTPG1, MAH1, MYB30/41/96, PAS2, W1N1/SHN1, WBC11*, and *WSD1* (Samuels et al., 2008; Bernard and Joubès, 2013; Lee and Suh, 2013) were mapped and cloned. Among them, *CER4, CER6, CER10, FATB, WSD1*, and *MAH1* were related to wax biosynthesis, while *CER5, CER7, WBC11, W1N1/SHN1, MYB30/41/96, CFL1*, and *HDG1* were responsible for the transportation and regulation (Samuels et al., 2008; Lee and Suh, 2013). Besides, *OsGL1-1* to *OsGL1-11, OsGL1, OsWSL2* and *OsWSL3* in rice (*Oryza sativa*) (Islam et al., 2009; Qin et al., 2011; Mao et al., 2012; Zhou et al., 2013; Gan et al., 2016), *ZmGL1, ZmGL2*, and *ZmGL8* in maize (*Zea mays*) (Tacke et al., 1995; Xu et al., 2002; Sturaro et al., 2005), *HvCUT1.1, HvCUT1.2, HvCUT1.3, HvFDH1.1, HvCER1.1*, and *HvCER1.2* in barley (*Hordeum vulgare*) (Richardson et al., 2007), and *W1, W2, Iw1, Iw2, Iw3, Wx-7A, Wx-4A, Wx-7D*, and *Wax1* in wheat (*Triticum aestivum* Linn.) (Dubcovsky et al., 1997; Murai et al., 1999; Lu et al., 2015) were mapped. Within *Brassica* species, *BnCER1* in canola (*Brassica napus*) (Pu et al., 2013) and *BrWax1* in Chinese cabbage (*Brassica rapa* L. ssp. *pekinensis*) (Zhang et al., 2013) were also mapped and characterized.

Among *Brassica* species, most studies focused on genetic analyses of waxy mutants. The glossy trait in broccoli (*Brassica oleracea* L. var. *italica* Plenck) (Anstey and Moore, 1954; Farnham, 2010), Brussels sprouts (*B. oleracea* L. var. *gemmifera* Zenk) (North and Priestley, 1962), and *B. napus* ‘Nilla glossy’ (Thompson, 1972; Jianguo et al., 1995) is controlled by recessive genes respectively, but dominant genes in *B. napus* wl mutant (Jianguo et al., 1992) and collard (*B. oleracea* L. var. *sabauda* DC.) (Priestley and Wills, 1966). However, no genes related to glossy trait in cabbage have been identified, and the molecular mechanism of cuticular wax biosynthesis and secretion in *B. oleracea* has yet to be fully characterized.

In this study, using simple sequence repeat (SSR) markers and two F_2_ populations, *Cgl1*, a gene controlling glossy trait in cabbage, was fine mapped in a 188.7 kb region at the end of chromosome C08. According to gene annotation and homology analyses, *Bol018504* was identified as the candidate gene of *Cgl1* from 33 genes in target region. Moreover, further analysis on coding and promoter sequences of *Bol018504* were made to validate the fine-mapping result. Findings in the present research will contribute to a more comprehensive understanding of plant cuticular wax metabolic networks and accelerate the breeding of cabbage cultivars exhibiting the brilliant green trait.

## Materials and Methods

### Plant Materials and Growth Conditions

**FIGURE 1:**
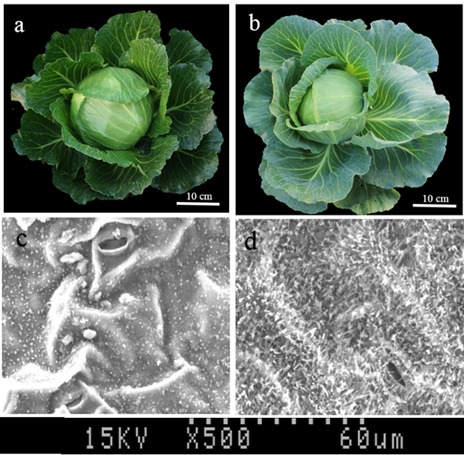
**Plant appearance of mutant 10Q-961, wild-type 10Q-962, and the micro-structure observation of leaf blade. (a)** Mutant 10Q-961, **(b)** wild-type 10Q-962, **(c)** microstructure of 10Q-961 blade ventral, **(d)** microstructure of 10Q-962 blade ventral.

Line 10Q-961 (a glossy mutant of inbred cabbage line10Q-962) (**Figure 1**), and the waxy inbred cabbage line 10Q-206 were used as parents to generate an F_1_ hybrids. The F_2_ population was generated through self-pollination of F_1_ plants. To acquire more polymorphism markers and accelerate the mapping process, another F_2_ population was produced from a cross between line 10Q-961 and a waxy Chinese kale doubled haploid line, M-36.

Plant phenotype was investigated visually at three-real-leaf stage. All plants were grown in spring and autumn each year in a solar greenhouse at the experimental station in Changping (39°54′ N, Beijing, China). All plant materials were provided by the Cabbage and Broccoli Research Group, Institute of Vegetables and Flowers, Chinese Academy of Agricultural Sciences.

### Map-Based Cloning of *Cgl1*

Genomic DNA from young leaves was extracted using CTAB method with modifications (Tel-Zur et al., 1999). For preliminary mapping, bulked segregant analysis (BSA) (Chantret et al., 2000) was performed with DNA pools from 10 waxy F_2_ individuals and 10 glossy F_2_ individuals. 189 glossy individuals of the F_2_ population which produced from a cross between line 10Q-961 and 10Q-206 were used in preliminary mapping. About 5, 000 F_2_ seeds (10Q-961 × M-36, self-pollination) were planted, and 1,172 individuals with a glossy phenotype were selected for fine mapping purposes.

### Simple Sequence Repeat Markers, Polymerase Chain Reaction, and Polyacrylamide Gel Electrophoresis

A total of 866 SSR markers evenly distributed on nine chromosomes were designed according to the whole genome sequence of *B. oleracea*^1^ (Liu et al., 2014). Primers were designed to produce 100–460-bp amplicons with 40–50% GC content and a melting temperature of 57–61°C.

A polymerase chain reaction (PCR) was completed in a 10 μl reaction volume containing 2 μl DNA template (50 ng μl^-1^), 1 μl 10× PCR buffer [200 mM Tris-HCl pH 8.3, 200 mM KCl, 100 mM (NH_4_)_2_SO_4_, 20 mM MgSO_4_], 0.8 μl dNTP (2.5 mM), 0.5 μl forward primer (5 μM), 0.5 μl reverse primer (5 μM), 0.2 μl Taq DNA polymerase (2.5 U μl^-1^), and 5 μl double-distilled H_2_O. The PCR conditions were as follows: initial denaturation at 96°C for 8 min, followed by 33 cycles of 94°C for 30 s, 55°C for 30 s, and 72°C for 40 s. The PCR program was completed with a final extension step at 72°C for 7 min. The PCR was conducted using a Veriti 96-Well Thermal Cycler (Applied Biosystems, Carlsbad, CA, USA). Amplicons were analyzed using 8% polyacrylamide gel electrophoresis at 160 V for 70 min, and visualized by silver staining (Bassam et al., 1991).

To analyze the *Bol018504* candidate gene, five primer pairs were designed using DNAMAN 7.0 based on the ORF and putative promoter sequence of *Bol018504*. A PCR amplification was completed using the Q5 Ultra High Fidelity DNA polymerase (New England Biolabs, Inc.) following its manufacturer’s instructions. Genomic DNA of line 10Q-961 and wild-type line 10Q-962 were used as PCR templates, and the resulting amplicons were analyzed by agarose gel electrophoresis.

### Quantitative Reverse-Transcription PCR (qRT-PCR) Analysis of the Candidate Gene

Total RNA was prepared from young leaves of 10Q-961 and 10Q-962 using a RNAprep pure Plant Kit (TIANGEN, Beijing, China), according to the manufacturer’s instructions. The extracted RNA was treated with RNase-free DNase I (Fermentas, Harrington, QC, Canada) to eliminate genomic DNA contamination. 1 μg of RNA was used to synthesize oligo (dT)-primed first-strand cDNA using a PrimeScript 1st Strand cDNA Synthesis Kit (TaKaRa, Kyoto, Japan) following the protocol provided by the manufacturer. Primer QRT504 was designed for quantitative analysis of *Bol018504*. Primer sequences of both actin and *Bol018504* used in qRT-PCR analysis are listed in **Table 1**. qRT-PCR was carried out using the SYBR^®^ Premix Ex Taq^TM^ II (Tli RNaseH Plus) kit (Takara) to analyze gene expression. Amplification was performed on a CFX96 Touch^TM^ real-time PCR detection system (Bio-Rad, Hercules, CA, USA). Three technical replicates were used for each cDNA sample and three samples (biological replicates) were tested. The relative mRNA expression was calculated using the 2^-ΔΔCt^ method (Livak and Schmittgen, 2001).

**Table 1:** The primer sequences information used in this paper.

Primer name	Forward (5′–3′)	Reverse (5′–3′)
LTSSR740	CCCTAAAGATCCGACAAGGC	ATCGTGGGAATAGAGGGCTT
C08SSR7	GTAATGCTGTTCCGTTGCAG	TCAGCATCAGAATGTGGCTC
C08SSR19	CAATTGAGTGGCCTTTTGGT	TAAAATCTTGGATCGGGGTG
C08SSR26	TACAAGGACCACCATGCTCA	CGCCATGAGTAACAGCTGAA
C08SSR46	CCATCCATCCGCTTGTAAAT	TCGTGAAGGGATGATGATGA
C08SSR53	GATCAATGCCAAACGGAGAG	ATCCTGATCAACGGAGCAAC
C08SSR54	GTGACCTGAGGAAGCAGAGC	GTCCCGGTTCAAGAAAACAA
C08SSR55	TCGTCCGTCATGTCATCATT	AGGAGAGTCGAGCACAAACC
C08SSR56	CACTAACGCTTTTTGACCCA	AGAAGCCAAGGACCATGCTA
C08SSR61	CTCCCGACTTCAGAAACTGC	TTGCCGTTGGATAAGGACTC
A	GCATAAGAAGGTGTGCCCT	CATTGCGGTTGCTACTGTC
B	CTCTCTGGACATAACCTCCC	CATAAAGCACAAGCGACG
C	TCGTATTGCCCTTCTTGC	CATTGTGCCGTAGATGTAGTCA
D	ACCTCTTTCCTCCACTCAAGT	CTATTTATCACAACGGCTGC
E	GATTGAGAAAGCGATACTGGAG	TGATAGGGTGGTTACCTGTCT
P504	GCATAAGAAGGTGTGCCCTG	GAGACAAAGAGGCTGGCGTA
504	CCACTTTCTTTACTCCCGCT	CGGATTTGTTTGGTGACTTG
ISP1	GGTTGGCTTCGTCATTCTA	GAACAGCAATCCGTTGAAC
QRT504	AGGACAGACGGAGTGTTGA	GGTAGCGGGAGTAAAGAAAGT
Actin	CCAGAGGTCTTGTTCC	GTTCCACCACTGAGCACAA
	AGCCATC	TGTTAC

### Data Analysis

For each marker, individuals with the 10Q-961 mutant allele were categorized as “b.” Individuals with the 10Q-206 or M-36 alleles were categorized as “a.” Those with the F_1_ allele were categorized as “h.” The Kosambi mapping function was used to calculate genetic distances between markers (Kosambi, 1943) and the genetic map was constructed using MapDraw (Liu and Meng, 2003).

## Results

### Preliminary Mapping of the *Cgl1* Locus

**FIGURE 2:**
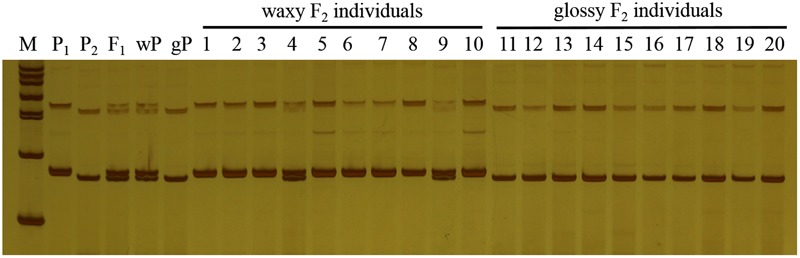
**Polymorphisms of LTSSR740 in parents, F_1_, gene pools, and partial individuals of the F_2_ population.** M, DNA ladder; P_1_, line 10Q-206 with waxy phenotype; P_2_, line 10Q-961 with glossy phenotype; F_1_, hybrid; wP, waxy pool; and gP, glossy pool. Lanes 1–10, waxy F_2_ individuals; lanes 11–20, glossy F_2_ individuals.

**FIGURE 3:**
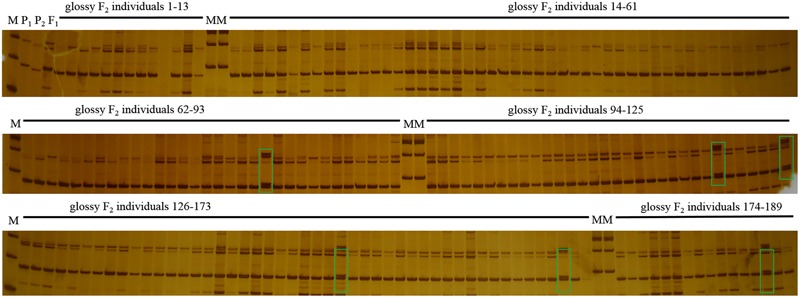
**Polymorphisms of LTSSR740 in parents, F_1_, and 189 individuals of the F2 population.** M, DNA ladder; P_1_, 10Q-206 with waxy phenotype; P_2_, 10Q-961 with glossy phenotype; F_1_, hybrid. Lanes 1–189, glossy F_2_ individuals. Green boxes represent recombinant plants.

In this study, F_1_ hybrids of 10Q-961 (P_1_) × M-36 (P_2_) were all exhibit waxy phenotype as M-36. In F_2_ population, the ratio of waxy to glossy plants was 3.085:1 (398:129), which was confirmed to be 3:1 by the Chi-square test (χ^2^ = 0.076 < χ^2^_0.05_ = 3.841). The ratio of waxy to glossy plants was 0.933:1 (236:253), which was confirmed to be 1:1 by the Chi-square test (χ^2^ = 0.591 < χ^2^_0.05_ = 3.841) in BC_1_P_1_ population, and all the plants in BC_1_P_2_ backcross population exhibited waxy phenotype. These genetic analysis indicated that the glossy traits in line 10Q-961 was controlled by a single recessive gene. 886 SSR primers were used to screen the polymorphisms between the parent lines 10Q-961 and 10Q-206. A total of 61 of the 866 primer pairs showed polymorphisms. Markers that were polymorphic between parents were screened in two bulks. Of all the primers, only marker LTSSR740 was identified polymorphisms between the two pools (**Figure 2**). The F_2_ population of the 10Q-961 × 10Q-206 cross consisted of 189 recessive plants and was used to validate the LTSSR740 marker. Six recombinants (**Figure 3**) were detected, and the calculated genetic distance between LTSSR740 and *Cgl1* was 3.17 cM. According to the location of LTSSR740 in the ‘02-12’ cabbage reference genome, *Cgl1* was localized to the end of chromosome C08 (40,596,166–41,516,064).

### Fine-Mapping of the *Cgl1* Locus and Candidate Gene Analysis

**FIGURE 4:**
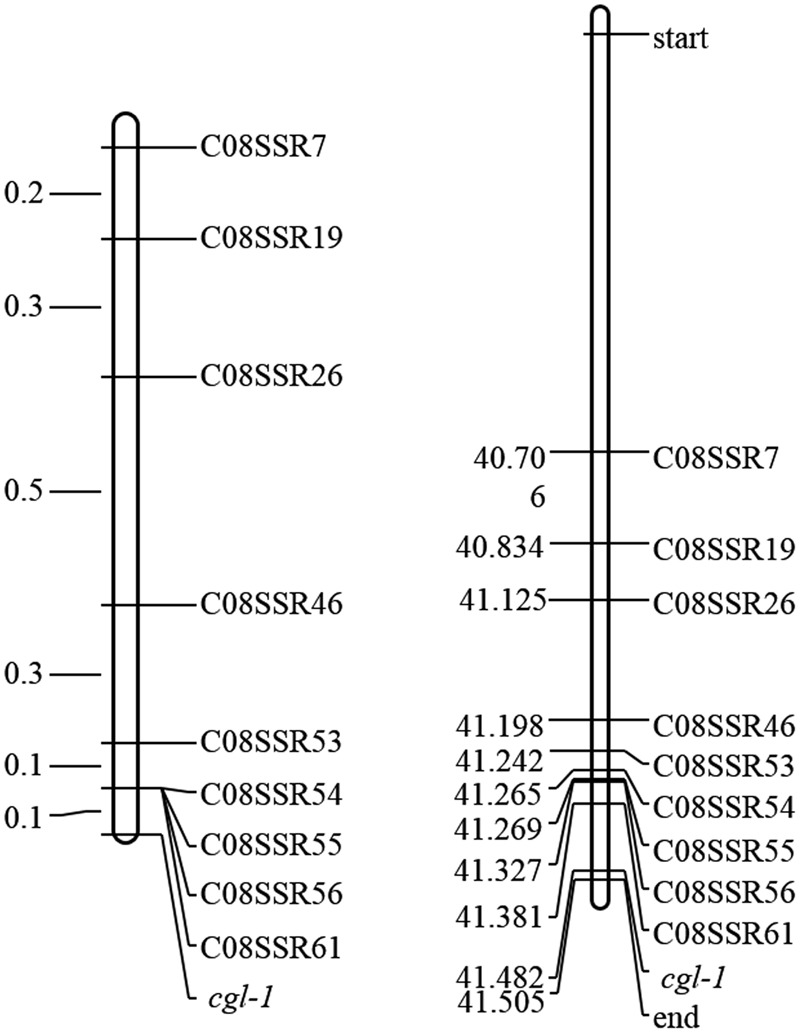
**Genetic map of *Cgl1* (**left**, unit: cM) and the corresponding physical map (**right**, unit: Mb)**.

To identify marker loci closely linked to *Cgl1*, 108 SSR markers were designed nearby marker LTSSR740. Nine of these markers, namely C08SSR7, C08SSR19, C08SSR26, C08SSR46, C08SSR53, C08SSR54, C08SSR55, C08SSR56, and C08SSR61 (**Table 1**), were polymorphic between lines 10Q-961 and M-36. These markers were used to screen 1,172 F_2_ individuals with the glossy phenotype, 15, 12, 6, 2, 1, 1, 1, 1, 1 recombinant plants were detected, respectively. This results indicated that *Cgl1* was located between C08SSR61 and the end of chromosome C08 (41,327,369–41,516,064, genetic and physical map distances of 0.085 cM and 188.7 kb, respectively). The order of the nine SSR markers in the genetic map was consistent with that of the physical map (**Figure 4**). According to ‘02-12’ genome reference^1^, 33 genes were located in this 188.7-kb region. Based on gene annotations of cabbage and alignments with *A. thaliana, Bol018504* was revealed to be highly homologous to *AtCER1* which encodes an aldehyde decarbonylase catalyzing the process of conversion from C30 aldehydes to C29 alkanes of wax synthesis pathway. Thus, we tentatively designated *Bol018504* as the candidate gene for the *Cgl1* locus.

### Verification and Expression Level of the *Bol018504* Candidate Gene

**FIGURE 5:**
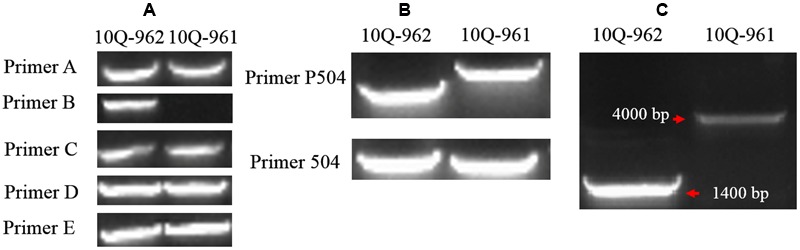
**Amplification products of each primer pair using templates from lines 10Q-962 and 10Q-961. (A)** Products of primer pairs A–E, **(B)** products of primer pairs P504 and 504, **(C)** products of primer pair B after the PCR extension time was increased.

Five primer pairs (A–E) were designed spanning the full length of *Bol018504* and its putative promoter region (**Tables 1** and **2**). There were no differences between the 10Q-961 and 10Q-962 amplification products generated from primer pairs A, C, D, and E. While primer pair B produced an approximately 1400-bp amplicon from the 10Q-962 template that was not amplified using the 10Q-961 template (**Figure 5A**). To avoid the possibility that primer pair B would not produce any amplicons from the 10Q-961 template, the full length *Bol018504* gene and its promoter region were divided into two fragments (**Table 2**). Two primer pairs (P504 and 504) were used to amplify DNA templates from mutant and wild-type plants. Polymorphisms were detected only when using primer pair P504 (**Figure 5B**). The sequencing of the PCR products of the two primer pairs revealed that the primer pair 504 amplicons were identical between lines 10Q-961 and 10Q-962, while the primer pair P504 amplicon in the glossy mutant had a 2,722-bp insertion that was absent in the wild-type amplification product. To eliminate the possibility that the elongation time (1.0 min) was insufficient for primer pair B amplification of 10Q-961 DNA, it was increased to 2.5 min. The resulting approximately 4000-bp amplification product for line 10Q-961 and approximately 1400-bp product for line 10Q-962 (**Figure 5C**), and further confirmed the presence of a 2,722-bp insertion in line 10Q-961 by Sanger sequencing.

**Table 2:** Primer amplification ranges and fragment lengths of *Bol018504.*

Primer	A	B	C	D	E	P504	504
Amplification range	-1777 to -865	-941 to 500	307 to 1754	1051 to 2451	2189 to 3672	-1777 to 1984	706 to 3898
Fragment length (bp)	912	1441	1447	1400	1483	3761	3192

*The adenine nucleotide of the ATG start codon was considered position 1.*

**FIGURE 6:**
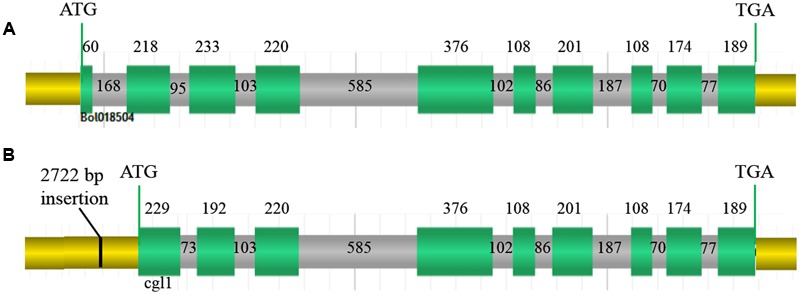
**Genomic organization. (A)** Genomic organization of *Bol018504* in wild-type line 10Q-962, **(B)** genomic organization of *Cgl1* in mutant line 10Q-961. Green sections, exons encoding amino acids; gray sections, introns; yellow sections, 5′- and 3′-untranslated regions; black line, 2,722-bp insertion. Numbers represent the lengths of each region in base pairs.

According to the FGENESH 2.6 prediction, the full-length *Bol018504* sequence in the wild-type line 10Q-962 (*CGL1*), comprising of 10 exons and nine introns (**Figure 6A**) was 3,351 bp, with coding sequence of 1,887 bp. While in the mutant line 10Q-961, *Cgl1*consisted of only nine exons and eight introns (**Figure 6B**), with ORF of 3,071 bp and coding sequence of 1,797 bp. The first exon of *CGL1* was absent in *Cgl1* and the positions of the second and third *CGL1* exons differed in *Cgl1*, indicating the 2,722-bp insertion changed the RNA splice site. Using the National Center for Biotechnology Information BLAST tools, alignment analysis of the predicted *Cgl1* amino acid sequence detected a missing PLN02869 domain, which controls fatty aldehyde decarbonylase activity. Thus, disruption of *Cgl1* may result in inhibited conversion of aldehydes to alkanes and cause the glossy phenotype in mutant cabbage lines.

**FIGURE 7:**
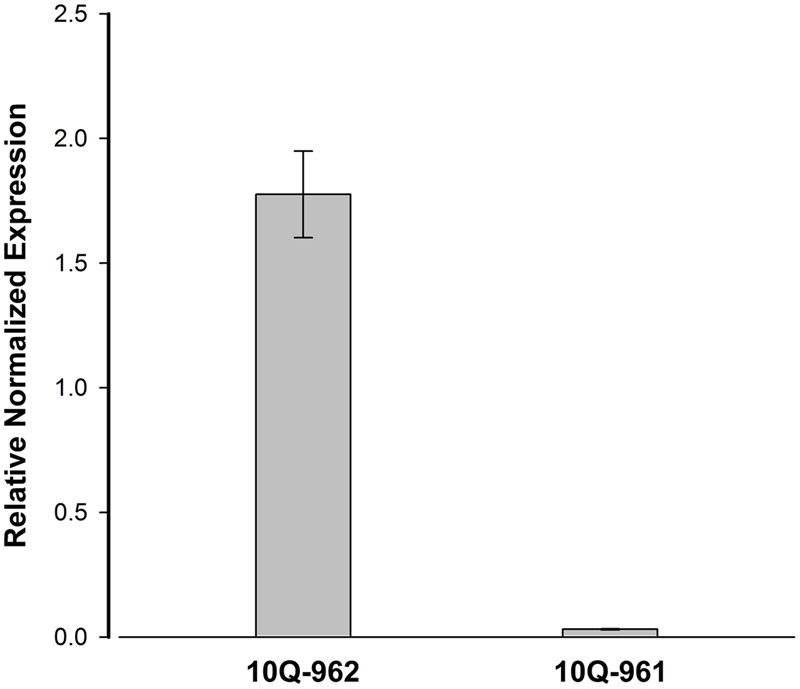
**Real-time PCR analysis for the expression patterns of *Bol018504* in mutant 10Q-961 and wild-type 10Q-962.** Data represent means and standard errors of three replicates.

The expression level of *Bol018504* between 10Q-961 and 10Q-962 were measured to determine whether the 2,722-bp insertion affected the expression of *Bol018504*. The expression levels of *Bol018504* in leaf of the two materials at three-leaf stage seedlings were measured (**Figure 7**), and expression of the candidate gene was about 57.1 times higher in wild type 10Q-962 compared to mutant type10Q-961.

### The 2,722-bp Insertion is Completely Linked with Mutant Phenotype in Recombinant Plants

**FIGURE 8:**
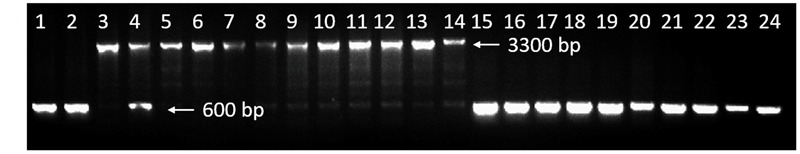
**Validation of ISP1 marker.** Lane 1, wild-type 10Q-962; lane 2, Chinese kale doubled haploid line, M-36; lane 3, mutant 10Q-961; lane 4, 10Q-961 × M-36 F1 hybrid; lanes 5–14, 10 recombinant plants in F2 mapping population; lanes 15–16, a dominant glossy mutant and its wild type; lanes 17–18, another recessive glossy mutant and its wild type; lanes 19–24, cabbage inbred lines 96–100, 87–534, xiafeng, mingde, Ikama and 8282.

Based on the insertion, a molecular marker, ISP1 (**Table 1**) which could amplified an approximately 3,300-bp and 600-bp amplification product from line 10Q-961 and 10Q-962 respectively was developed to analyze the recombinant plants. We found that the 2,722-bp insertion was completely linked with the mutant phenotype in the F_2_ mapping population (**Figure 8**). To determine whether this insertion was conserved in different varieties, a dominant glossy mutant and its wild type, another recessive glossy mutant and its wild type and six cabbage inbred lines available in our laboratory were tested. Agarose gel electrophoresis suggested that ISP1 marker produced approximately 600-bp amplicon in these 10 cabbage materials (**Figure 8**). This result indicated that the 2,722-bp insertion was conserved in glossy mutant 10Q-961, and that the ISP1 marker can distinguish the glossy mutant 10Q-961 from the other cabbage.

## Discussion

Our previous study revealed that the glossy trait in line 10Q-961 was controlled by a single recessive gene (Li et al., 2012). This result was also confirmed in this study. In the current report, 1,361 recessive glossy individuals from two different F_2_ populations were used to map the *Cgl1* gene. Using a standard molecular genetic mapping strategy, *Cgl1* was localized to a 188.7-kb region between the C08SSR61 SSR marker and the end of chromosome C08. Sequence and gene annotation analyses revealed that the *Bol018504* candidate gene was highly homologous to *AtCER1*, which encodes an enzyme that catalyzes the presumed decarbonylation of aldehydes to alkanes (Aarts et al., 1995).

Our previous study involving scanning electron microscopy and gas chromatography-mass spectrometry analyses reported a decreased abundance of cuticular waxes in mutant line 10Q-961, with only 30.57% of the total wax content of the wild-type line 10Q-962; additional studies concluded that the reduced cuticular wax content in the mutant lines was mainly due to a decreased amount of C_29_ alkane; the lower C_29_ alkane levels were accompanied by a slightly increased C_30_ aldehyde content (Tang et al., 2015). These results were similar to those for the *A. thaliana cer1-1* mutant (Bourdenx et al., 2011). In this study, the disruption of *Bol018504* in mutant line 10Q-961 was the likely cause of its glossy phenotype. These results suggest that the cabbage *CGL1* gene has the similar function as its *A. thaliana* homolog *CER1*.

According to comparative genome mapping, a triplication process occurred during cabbage evolution. Thus, an *A. thaliana* gene fragment may correspond to approximately three homologous copies in *Brassica* species (Cheng et al., 2014; Liu et al., 2014). There are two *AtCER1* homologs in cabbage, namely *Bol018504* and *Bol035365*. The *Bol035365* fragment is an incomplete copy, with no sequence differences between mutant line 10Q-961 and wild-type line 10Q-962 (data not shown). Whether this gene is essential for CER1 protein function in cabbage is unclear. In this study, a 2,722-bp insertion in the first intron of *Bol018504* may be responsible for the glossy mutant phenotype.

*Brassica oleracea* encompasses multiple cultivar groups that are classified based on the specialized morphology of their edible structures, namely kales (*B. oleracea* var. *sabellica*), cabbages (*B. oleracea* var. *capitata* f, *alba*), broccoli (*B. oleracea* var. *italica*), cauliflower (*B. oleracea* var. *botrytis*), brussels sprouts (*B. oleracea* var. *gemmifera*), kohlrabi (*B. oleracea* var. *gongylodes*), collard greens (*B. oleracea* var. *viridis*), savoy cabbage (*B. oleracea* var. *capitata* f, *sabauda*), romanesco broccoli (*B. oleracea* var. *botrytis*), broccolini (*B. oleracea* var. *italica* × *alboglabra*), and kai-lan (*B. oleracea* var. *alboglabra*). *Brassica* research has been enhanced through the publication of the *B. rapa* genome sequence and *B. napus* genome sequence (Wang et al., 2011; Chalhoub et al., 2014). Homolog sequences of this 2,722-bp insertion have been identified in Chinese cabbage (*B. rapa*; A09: 31,590,811–31,593,425) and canola (*B. napus*; C08: 38,256,925–38,259,552), but only the last 719 bp of the insertion is present in the cabbage genome (*B. oleracea* L. var. *capitata*; C02: 16,412,989–16,413,707). However, a 2,681-bp homolog sequences (C02: 7,222,204–7,224,885) of this 2,722-bp insertion could been identified in a doubled haploid *B. oleracea* kale-like type TO1000DH genome (Parkin et al., 2014). The current research has proved the existence of this 2,722-bp sequences in the wild-type 10Q-962, low sequence alignment against cabbage genome may be caused by sequencing or splicing error in cabbage genome assembly. Thus, further perfection of the cabbage genome sequences is supposed to be conducted.

A molecular marker, ISP1 which could distinguish the glossy mutant 10Q-961 from the other cabbage was developed. This molecular marker could be used for marker assistance selection in cabbage breeding and accelerate the breeding process of bright green cabbage. Additionally, few seeds were produced after self-pollination of 10Q-961 mutant line, which is consistent with observations for the *cer1 A. thaliana* mutant (Aarts et al., 1995). Future studies will be completed to determine whether *Cgl1* influences fertility of cabbage.

To the best of our knowledge, *CGL1* is the first wax synthesis gene mapped in *B. oleracea*. This achievement is an important advance for molecular research on wax synthesis in cabbage. Future functional studies on this gene will contribute to a more comprehensive understanding of plant cuticular wax metabolic networks.

## Author Contributions

ZLiu developed the F_2_ populations, performed the experiments, analyzed the data, wrote and revised the manuscript. ZLiu, DL, and JT isolated the samples. LY, ZF, YL, MZ, YZ, ZZ, PS, HL, and ZLi conceived of the study and critically reviewed the manuscript. ZLiu and JT analyzed the sequencing data and designed the SSR primers. All authors read and approved the final manuscript.

## Conflict of Interest Statement

The authors declare that the research was conducted in the absence of any commercial or financial relationships that could be construed as a potential conflict of interest.

## Footnotes

1 http://brassicadb.org
